# Potentiation of photodynamic therapy of cancer by complement: the effect of *γ*-inulin

**DOI:** 10.1038/sj.bjc.6603508

**Published:** 2006-12-05

**Authors:** M Korbelik, P D Cooper

**Affiliations:** 1 British Columbia Cancer Agency, Vancouver, BC, Canada V5Z 1L3; 2 Tumor Biology Group, Australian National University Medical School and The Canberra Hospital, Woden, ACT 2603, Australia

**Keywords:** photodynamic therapy, complement system, *γ*-inulin, CD 8 lymphocytes, mouse tumours

## Abstract

Host response elicited by photodynamic therapy (PDT) of cancerous lesions is a critical contributor to the clinical outcome, and complement system has emerged as its important element. Amplification of complement action was shown to improve tumour PDT response. In search of a clinically relevant complement activator for use as a PDT adjuvant, this study focused on *γ*-inulin and examined its effects on PDT response of mouse tumours. Intralesional *γ*-inulin (0.1 mg mouse^−1^) delivered immediately after PDT rivaled zymosan (potent classical complement activator) in delaying the recurrence of B16BL6 melanomas. This effect of *γ*-inulin was further enhanced by IFN-*γ* pretreatment. Tumour C3 protein levels, already elevated after individual PDT or *γ*-inulin treatments, increased much higher after their combination. With fibrosarcomas MCA205 and FsaR, adjuvant *γ*-inulin proved highly effective in reducing recurrence rates following PDT using four different photosensitisers (BPD, ce6, Photofrin, and mTHPC). At 3 days after PDT plus *γ*-inulin treatment, over 50% of cells found at the tumour site were CTLs engaged in killing specific targets via perforin–granzyme pathway. This study demonstrates that *γ*-inulin is highly effective PDT adjuvant and suggests that by amplifying the activation of complement system, this agent potentiates the development of CTL-mediated immunity against PDT-treated tumours.

Photodynamic therapy (PDT), in which the destruction of cancerous lesions is achieved by the targeted site-localised generation of reactive oxygen species formed by the transfer to molecular oxygen of energy captured by the light excitation of photosensitising drugs (Dougherty *et al*, 1998; Huang, 2005), has not yet reached its full clinical potential. In addition to further development of more potent photosensitising drugs and their selective tumour localization, a promising approach appears in exploiting the PDT-associated host response. Tumour PDT elicits a strong host response orchestrated by the innate immune system that culminates in the development of adaptive immunity against the treated lesion, and renders an important contribution to the therapy outcome (Henderson and Gollnick, 2003; Castano *et al*, 2006; Korbelik, 2006). It has recently become evident that complement system is engaged at multiple levels in the execution of this host response, including (i) initial recognition of endogenous danger signals generated by tumour-localised insult inflicted by PDT, (ii) incitement and propagation of the elicited inflammatory response, (iii) efferocytosis (dead cell removal), and (iv) tumour antigen presentation and promotion of adaptive immune response recognising the PDT-treated tumour as its target (Cecic and Korbelik, 2006; Cecic *et al*, 2006; Korbelik and Sun, 2006).

Supportive evidence of the importance of the contribution of the activated complement system includes the decrease in cure rates of PDT-treated tumours following blockage of complement activation by the inhibitor FUT-175 (Korbelik, 2006), or specific inactivation of complement anaphylatoxins C3a and C5a (Cecic *et al*, 2005). On the other hand, enhancement of complement activation following tumour PDT was shown to boost the curative outcome (Korbelik *et al*, 2004). This has led to the idea to exploit the involvement of complement system in PDT response by amplifying its activity with adjuvant treatment for stimulating complement activation. In initial studies that established the potential of such adjuvants for increasing the effectiveness of tumour PDT, we used zymosan, streptokinase, and urokinase (Korbelik *et al*, 2004). Zymosan (particles from the cell wall of *Saccharomyces cerevisiae*) is a standard and potent complement activator but it is not safe for clinical use (Volman *et al*, 2002), while streptokinase and urokinase are clinically acceptable but are primarily acting as anticoagulating agents (Ewald and Eisenberg, 1995; Oren *et al*, 1998). Hence, the present study evaluated *γ*-inulin as the adjuvant to tumour PDT because this neutral polyfructose derived from storage carbohydrates of *Compositae* plant family is highly potent in specific activation of the alternative pathway of complement system and has established safety for human use (Frazer *et al*, 1999). Highly successful Phase I clinical trial with *γ*-inulin as adjuvant and hepatitis B surface antigen is just completed, with no safety issues and good human activity (Petrovsky N *et al*, unpublished data). The present study reports that *γ*-inulin treatment is highly effective in elevating PDT-mediated cures of mouse tumours and reveals that its action amplifies CTL activity against PDT-treated tumours.

## MATERIALS AND METHODS

### Tumour models

Melanoma B16BL6 (Poste *et al*, 1980) and fibrosarcoma MCA205 (Barth *et al*, 1990) were grown in syngeneic C57BL/6 mice, and fibrosarcoma FsaR (Volpe *et al*, 1985) in syngeneic C3H/HeN mice using 6–8-week-old females. The mice were kept in sterile cages housed in the animal facility of the British Columbia Cancer Research Centre. All animal procedures were conducted according to the approval from The Animal Ethics Committee of the University of British Columbia and meet the standards required by the UKCCR guidelines (Workman *et al*, 1998). Cohorts of tumours for individual experiments were implanted from the same tumour cell suspension by injecting 1–2 × 10^6^ cells mouse^−1^ subcutaneously at the sacral region on the dorsal site. The tumours were treated when their largest diameter reached 6–8 mm (around 8 days post implant).

### *γ*-Inulin

The *γ*-inulin preparation was made from dahlia tubers as endotoxin-free suspension of 1–2 *μ*m ovoids of insoluble form of inulin, *β*-D-(2 → 1) polyfructofuranosyl *α*-D-glucose, in which an unbranched chain of up to 100 fructose moieties is linked to a single terminal glucose (Cooper and Carter, 1986a). The formulation selects the high molecular weight form (up to 16 000) of this inert polysaccharide with the greatest potency for the activation of alternative complement pathway by providing an optimised surface that binds C3b while shielding it from inactivation by factor H (Cooper, 1993). The treatments were performed by intratumoural injection of 0.1 mg (40 *μ*l) of *γ*-inulin suspension immediately after PDT light exposure. Controls involving the intratumoural injection of the same volume of saline verified the absence of injection volume-related unspecific effects.

### Photodynamic therapy treatment

The photosensitisers used in this study were benzoporphyrin derivative monoacid ring A (BPD or verteporfin, a lipid-formulated photosensitiser provided by QLT, Inc., Vancouver, BC, Canada), Photofrin (provided by Axcan Pharma, Inc., Mont-Saint-Hilaire, Quebec, Canada), m-tetrahydroxyphenylchlorin (mTHPC, received from Scotia Pharmaceuticals Ltd, Stirling, UK), and chlorin e6 (ce6, Frontier Scientific, Inc., Logan, UT, USA). They were injected intravenously at 24 h (Photofrin, mTHPC), 3 h (BPD), or 2 h (ce6) before photodynamic light treatment.

The procedure and equipment used for photodynamic light treatment was described in detail elsewhere (Cecic *et al*, 2005). Briefly, light for superficial tumour illumination was generated by a 150 W QTH lamp-based high throughput source with integrated ellipsoid reflector (Sciencetech Inc., London, Ontario, Canada) using interference filters matching the absorption peaks of individual photosensitisers. Delivered light fluence rate was 80–90 mW cm^−2^ (power output measured by a light power metre divided by the surface of the illuminated area encompassing the treated tumour). During light treatment, mice were restrained unanaesthetised in special holders designed to expose the sacral region of their backs.

For zymosan treatment, particles derived from the cell wall of *S. cerevisiae* (Z42500, Sigma Chemical Co., St Louis, MO, USA) were suspended in PBS and injected intratumourally (0.5 mg mouse^−1^, 50 *μ*l) immediately after PDT light treatment. Mouse IFN-*γ* (PeproTech Inc., Rocky Hill, NJ, USA) was administered peritumourally (3 × 10^4^ U mouse^−1^) at 24 h before PDT light treatment.

After therapy, mice were monitored for tumour growth and no sign of palpable tumour at 90 days post treatment qualified as a cure. The effect of adjuvants in the absence of PDT was tested by following tumour growth rates determined by caliper measurement of the lesion's three orthogonal diameters. Saline only controls were tested for all of the used agents and they verified the absence of unspecific effects related to injections.

### C3 enzyme-linked immunosorbent assay

Mice were killed and tumours excised at 2 or 6 h after PDT and/or *γ*-inulin treatment. The content of complement C3 protein in tumour tissue homogenates was determined using enzyme-linked immunosorbent assay (ELISA) assay as previously described in full detail (Cecic *et al*, 2005). Briefly, multiplate wells were coated with goat F(ab’)2 fragment to mouse C3 (Cappel Laboratories, Durham, NC, USA; 1 : 1000 dilution in PBS), and test samples were added after blocking procedure for a 1.5 h incubation at 37°C, which was followed by staining with HRP-conjugated goat anti-mouse C3 (Cappel; 1 : 5000 dilution in PBS with 0.05% Tween-20 and 1% normal goat serum). The mouse C3 for standard was isolated and purified from the plasma of male DAB/2J (C5-deficient) mice.

### Flow cytometry

Tumours excised at 3 days after *γ*-inulin only treatment, control untreated tumours, and tissue materials collected from the tumour sites at 3 days after PDT and PDT plus *γ*-inulin treatments (there was no sign of tumour left at that time with these two treatment groups) were subjected to standard enzymatic disaggregation procedure (Korbelik, 1993). Single-cell suspensions obtained in this way were stained with FITC-conjugated rat-anti-mouse CD107a (sc-19992) and PE-conjugated rat anti-mouse CD8 (both from Santa Cruz Biotechnology, Inc., Santa Cruz, CA, USA), plus PerCP-Cy5.5-conjugated rat anti-mouse panleukocyte marker CD45 (Pharmingen, BD Biosciences, Mississauga, Ontario, Canada). In addition, FITC-conjugated rat anti-mouse IgG_2a_ (eBioscience Inc., San Diego, CA, USA) was used as isotype control for CD107a. These samples were then analysed by three-colour flow cytometry using the Coulter Epics Elite ESP (Coulter Electronics, Hialeah, FL, USA), with 20 000 cells included for each test. The percentage of FITC-positive cells was determined in populations gated as CD45^+^CD8^+^.

### Statistical analysis

Tumour response/cure results were statistically analysed using Log-rank test. The differences between means of the data from other experiments were statistically evaluated based on Mann–Whitney test. Statistical differences were considered significant when *P*<0.05.

## RESULTS

### *γ*-Inulin as photodynamic therapy adjuvant with B16BL6 tumours

**Figure 1:**
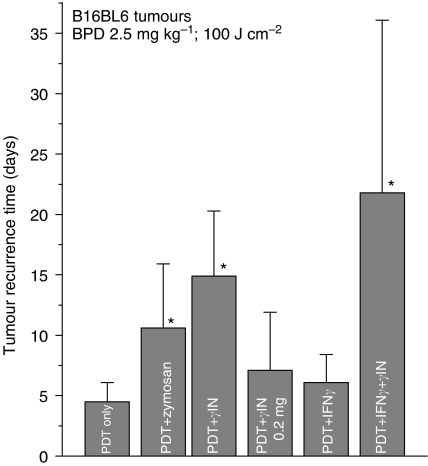
The effect of *γ*-inulin and zymosan on PDT response of B16BL6 tumours. Subcutaneously growing tumours were treated by PDT (BPD 2.5 mg ml^−1^ i.v. followed 3 h later by 100 J cm^−2^ of 690±10 nm light). Zymosan (0.5 mg mouse^−1^) and *γ*-inulin (0.1 mg mouse^−1^ except with one treatment group that received 0.2 mg mouse^−1^) were administered by intratumoural injection immediately after PDT light treatment, whereas IFN-*γ* (3 × 10^4^ U mouse^−1^) was given peritumourally at 24 h before PDT light. All treated tumours showed initially a complete response followed by the recurrence after time intervals that varied with different treatment groups (presented in the ordinate as average time for tumour recurrence). There was one cured tumour in PDT+IFN-*γ*+*γ*-inulin group (which translates into 12.5% cure rate); for including it into the recurrence time calculation, it was assigned time of 55 days. *N*=8; bars are s.d. ^*^Statistically significant difference compared to PDT only group.

Mouse B16BL6 melanoma is one of the most PDT-resistant tumour models. With the use of BPD as photosensitiser for PDT, an initial ablation of subcutaneous tumours can be achieved within 1 day of treatment, but this is followed by the recurrence of these lesions within several days after treatment (Figure 1). Higher doses of photosensitiser and light than used in our experiments are not helpful because they are toxic to mice. However, improved PDT responses of these tumours can be obtained by adjuvant treatments with agents that specifically activate complement system. A single intratumoural injection of the classical complement activator zymosan (0.5 mg mouse^−1^) given immediately after PDT light treatment doubled the time for tumour recurrence, while over three-fold increase in the recurrence time was observed with *γ*-inulin (0.1 mg mouse^−1^) applied in the same fashion (Figure 1). Both agents were most effective when injected directly into the tumour. Zymosan or *γ*-inulin treatment without PDT showed no detectable impact on tumour growth (not shown). No benefit was found with increasing the *γ*-inulin dose in conjunction with PDT treatment to 0.2 mg per mouse (Figure 1). A potential for further enhancement of the effect of *γ*-inulin as PDT adjuvant may be found in adding IFN-*γ* (a single peritumoural injection of 3 × 10^4^ units per mouse at 24 h before PDT light treatment) to the treatment protocol. This IFN-*γ* treatment, which by itself was not effective in boosting PDT response, produced in the combination with *γ*-inulin on average over a four-fold prolongation in the post PDT recurrence time of B16BL6 tumours (Figure 1). Moreover, one among the eight tumours in this treatment groups was declared cured (no sign of recurrence within 90 days) and was assigned the recurrence time of 55 days in the calculation of the tumour recurrence interval for this group. This was the main reason for the large s.d. value for PDT+IFN-*γ*+*γ*-inulin group because of which the difference with PDT+*γ*-inulin has not reached the statistical significance. It should be noted that B16BL6 melanomas extensively metastasise from the primary site and form detectable lung metastases within 2 weeks of initial implantation. In the course of this study, there were in total 20 mice with tumour recurrence times longer than 2 weeks after PDT plus *γ*-inulin treatment and none of them showed when killed signs of macroscopic lung metastases. However, there were two cases of mice with no signs of local tumour recurrence that had to be killed at 4–5 weeks post therapy because they became affected with disseminated lung deposits.

### Tumour C3 levels

**Figure 2:**
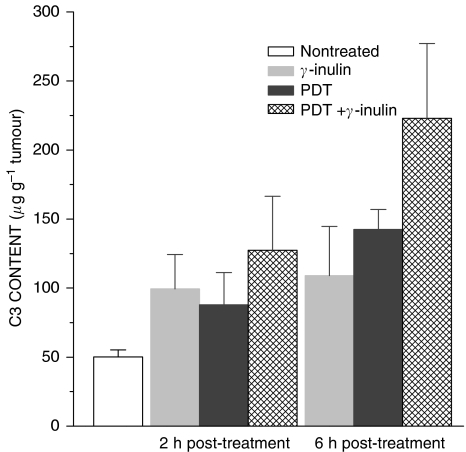
Complement C3 levels in B16BL6 tumours following treatment with PDT, *γ*-inulin, or their combination. Tumours were excised at either 2 or 6 h after treatments performed as described for Figure 1 (0.1 mg mouse^−1^ for *γ*-inulin), and samples from tumour tissue homogenates analysed by C3 ELISA. *N*=4; bars are s.d. Values for all 2 and 6 h post-treatment groups were statistically different than with nontreated group, and for PDT + *γ*-inulin group statistically higher than the other two groups at the 6-h time point.

To verify the impact of *γ*-inulin on complement activity in the chosen experimental model, B16BL6 tumours were excised at either 2 or 6 h after treatment and samples prepared by homogenisation of tumour tissue for ELISA-based determination of the levels of C3 protein (key component of the complement cascade). The results show that *γ*-inulin treatment alone produced a rise in tumour C3 levels at 2 h after injection with a similar level found 4 h later (Figure 2). A comparable elevation in tumour C3 levels was detected at 2 h after PDT only treatment, but an additional rise was evident at 6 h post PDT. Combining *γ*-inulin treatment with PDT showed no significant effect at 2 h post therapy, but produced significantly higher tumour C3 levels than *γ*-inulin or PDT alone (*P*<0.0286) at 6 h post therapy.

### Testing with various photosensitisers and tumour models

**Figure 3:**
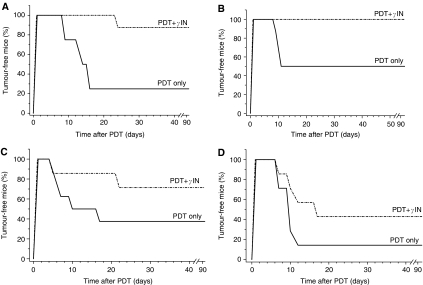
The effect of *γ*-inulin on PDT response of MCA205 and FsaR fibrosarcomas. Subcutaneously growing MCA205 fibrosarcomas were treated with (**A**) BPD-PDT (as described for Figure 1), (**B**) Photofrin-PDT (Photofrin 7.5 mg kg^−1^ i.v. followed 24 h later by 150 J cm^−2^ of 630±10 nm light), and (**C**) ce6-PDT (ce6 5 mg kg^−1^ i.v. followed 2 h later by 50 J cm^−2^ of 665±10 nm light), while (**D**) FsaR fibrosarcomas were treated with mTHPC-PDT (mTHPC 0.1 mg kg^−1^ i.v. followed 24 h later by 25 J cm^−2^ of 655±10 nm light). The treatment with *γ*-inulin (0.1 mg mouse^−1^) was as described for Figure 1. Following treatment (that resulted in an initial complete response), the mice were monitored for signs of tumour regrowth; no sign of tumour at 90 days post therapy qualified as a cure. *N*=8. In all four cases, there was statistically significant difference between responses of PDT+*γ*-inulin and PDT only groups.

The efficacy of *γ*-inulin as adjuvant to PDT was further examined with different tumour models and photosensitisers. The treatment of MCA205 fibrosarcomas with BPD-based PDT initially ablated all the lesions, but this was in most cases followed by the recurrence within the next 2 weeks and only 25% of mice remained tumour-free at 90 days post therapy (Figure 3A). The group receiving *γ*-inulin (0.1 mg mouse^−1^ i.t.) immediately after photodynamic light treatment showed a dramatic improvement evident as a decreased tumour recurrence compared to the PDT only group and high levels of cures. Similar results with the same tumour model were obtained with two different photosensitisers, Photofrin and ce6; in both cases, the chosen PDT only dose was in the moderate/halfway cure range and the adjuvant *γ*-inulin treatment increased the level of tumour cures to fully or highly curative range by reducing the recurrence rate (Figure 3B and C). This effect was also confirmed with a different syngeneic tumour model (subcutaneous FsaR fibrosarcomas growing in C3H/HeN mice) and PDT based on another photosensitiser (mTHPC) where *γ*-inulin elevated the tumour response rate from low to halfway curative range (Figure 3D). Similarly as with other tumour models, *γ*-inulin treatment in the absence of PDT had no detectable effect on FsaR tumours (not shown).

### Detection of CD107a-positive CTL

**Figure 4:**
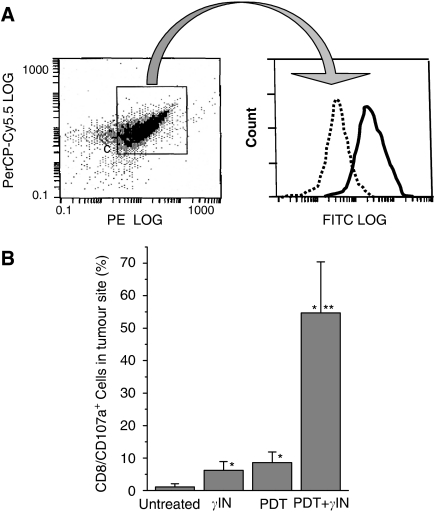
Detection of degranulating cytotoxic T lymphocytes at the site of tumour treatment with PDT, *γ*-inulin, or their combination. Subcutaneous MCA205 tumours were treated with ce6-PDT and/or *γ*-inulin as described for Figure 3. At 3 days after therapy, tumours or remnants collected at the treatment site were taken for preparing single-cell suspensions that were stained with antibodies against CD107a, CD8, and CD45 antigens and analysed by flow cytometry. (**A**) Representative example of staining of a PDT+*γ*-inulin sample showing FITC fluorescence intensity in a.u. per cell (CD107a and its isotype control) of the gated population staining positively for PerCP-Cy5.5 (CD45) and PE (CD8) and (**B**) percentage of CD107a-positive cytotoxic T lymphocytes in total cellular populations detected in different treatment groups. *N*=4; bars are s.d. ^*^Statistically significant difference compared to untreated tumours group; ^**^statistically significant difference compared to *γ*-inulin only and PDT only groups.

An insight into the mechanisms of the observed adjuvant effect of *γ*-inulin is revealed by the flow cytometry-based detection at the treated tumour site of cytotoxic T cells that have lysosomal-associated membrane protein-1 (LAMP-1, CD107a antigen) detectable on their surface (CD8^+^CD107a^+^), which occurs when these cells undergo degranulation (releasing granzyme B and perforin) upon their contact with specific target cells (Burkett *et al*, 2005). Very few, if any, such cells were found in untreated MCA205 tumours. At 3 days after either intratumoural *γ*-inulin injection or ce6-PDT only treatment, small but significant increases in the numbers of these cells were detectable at the treatment site. No evidence of palpable tumour was detectable at this time point with either PDT only or PDT plus *γ*-inulin treatment. With the latter group, the majority of cells collected from the treatment site were leukocytes and stained as CD8 positive (Figure 4A). Further gating of this CD8^+^ population revealed a high intensity of CD107a staining (in contrast to isotype control for this antibody) giving on average over 50% degranulating cytotoxic T cells per total collected cell population (Figure 4B).

## DISCUSSION

Nonantigenic and nontoxic agent *γ*-inulin is known as a specific activator of alternative complement pathway in humans, mice, and other mammalian species, and was demonstrated to be more potent than strong standard complement activators zymosan and killed *S. aureus* (Cooper and Carter, 1986a). Treatment of mice with *γ*-inulin was shown to result in deposition of complement C3-fragments on the surface of macrophages, which enables these antigen-presenting cells (APCs) to greatly increase their efficacy in enhancing the proliferation of antigen-specific T cells (Kerekes *et al*, 2001). Gamma-inulin is also a powerful adjuvant of the antibody responses (Cooper and Steele, 1988; Silva *et al*, 2004). Owing to these properties, there is also interest in *γ*-inulin as vaccine adjuvant, its safety for human use has been verified (Frazer *et al*, 1999). It has also been known for some time that intralesional *γ*-inulin treatment elicits antitumour responses and it was demonstrated that this results from the complement-activating effect (Cooper and Carter, 1986b). Positive responses following multiple treatment regimens were reported with mouse tumours (B16 melanoma model), squamous cell carcinoma in sheep, equine sarcoids, and spontaneous malignancies in dogs (Cooper and Carter 1986b; Cooper, 1993).

Our recent related work has shown that complement component C3 and other complement proteins accumulate in tumours treated by PDT (Cecic and Korbelik, 2002; Cecic *et al*, 2005), and that complement system recognises PDT-treated tumour cells as their target (Cecic *et al*, 2006). Binding of complement proteins to tumour antigens enhances their capture, processing, and presentation to T and B lymphocytes (Arvieux *et al*, 1988, Carroll, 2004), and with PDT-generated cancer vaccines this property appears critically important for securing ardent immune recognition of targeted tumour and the development of effective antitumour adaptive response (Korbelik and Sun, 2006).

Recurrence of tumours showing initially complete responses to PDT treatment has a major impact on the success or failure with this modality, and PDT-associated host response has become recognised as a key determinant of the recurrence rate of these tumours (Korbelik, 2006). The results of the present study demonstrate that the adjuvant treatment with *γ*-inulin is highly effective in reducing the recurrence rate of various types of mouse tumours following PDT mediated by different types of photosensitisers, including tumour vasculature-concentrated BPD and ce6, as well as tumour parenchyma-concentrated Photofrin and mTHPC. The obtained findings suggest that combining *γ*-inulin treatment with PDT secures fully or highly curative results with tumours that are not exceptionally resistant to PDT. Although in a direct comparison with the potent complement activator zymosan, using B16BL6 melanoma model, *γ*-inulin has not statistically proven significantly more effective in delaying the recurrence of PDT-treated tumours, it produced on average over three-fold increase in tumour recurrence time (compared to PDT only group) while this increase with zymosan was only two-fold (Figure 1). Moreover, curative threshold can be reached even with this highly PDT-resistant model by including the administration of IFN-*γ* before PDT plus *γ*-inulin treatment. The Th1 cytokine IFN-*γ* (originally called macrophage-activating factor) is well recognised for its capacity to stimulate phagocytic activity of macrophages and dendritic cells, and for augmenting the ability of these cells for antigen processing and presentation by increased quantity and diversity of peptides presented on their surface in the context of MHC (Schroder *et al*, 2004). Thus, IFN-*γ* treatment can boost the activity of macrophages and/or dendritic cells in securing, in conjunction with *γ*-inulin potentiated complement activation, immune recognition of PDT-treated tumours and augment immune rejection of these lesions with resultant prevention of tumour recurrence.

The assumed mechanism of action of *γ*-inulin as adjuvant to PDT, suggesting that reduced tumour recurrence rates are due to an enhanced activation of adaptive immunity, implies that increased numbers of cytotoxic T cells recognising cancer cells from the PDT-treated tumour as their target should be engaged at the treatment site. This was directly confirmed by the finding with PDT plus *γ*-inulin group that over 50% of cells at the tumour site at 3 days post PDT are CD107a-positive CD8 cells (Figure 4). These CD8 cells could express the CD107a antigen on their surface only after exocytosis of their granzyme and perforin-rich granules, which occurs upon attacking their specific targets in an antigen-specific manner (Betts *et al*, 2003).

Although detailed analysis of the impact of tumour-localised *γ*-inulin treatment on the formation of distant metastases was not the objective of this study, evidence was collected suggesting that this agent in conjunction with PDT attenuates metastatic spread of B16BL6 melanoma and this supports the existence of a systemic antitumour immune response developed after PDT plus *γ*-inulin treatment.

In summary, the present study demonstrates that the specific potent complement activator *γ*-inulin (nonantigenic natural carbohydrate verified as safe for human use) is a highly efficient adjuvant to tumour PDT with mouse tumour models. As its adjuvant efficacy was documented with different tumour types, including poorly immunogenic melanoma model, it can be expected that *γ*-inulin will work as adjuvant to PDT of human cancers. The results suggest that by boosting the activation of complement system, this agent potentiates the development of cytotoxic T-cell-mediated immunity against the PDT-treated tumour, which results in the abolishment or reduction in tumour recurrence rates.
